# Miscellaneous Notices

**Published:** 1845-06

**Authors:** 


### Miscellaneous Notices.

*Sixth Annual Meeting of the American Society of Dental Surgeons.*—The time for the sixth annual meeting of the American Society of Dental Surgeons, is now near at hand. Before another number of the Journal shall have been issued, it will have taken place, and we hope to have the pleasure of seeing a full attendance of the members on the occasion. We have recently learned that one residing in Havana, expects to be present. At past meetings, there have been members in attendance from the remotest parts of the Union. Such examples of sacrifice of time and money, reflect upon those who have made them the highest praise, and are worthy of general imitation. No member should be absent from the meetings of the society, when it is possible for him to be present.

Of the benefits to be derived from attendance on these meetings, we have frequently spoken before. We shall, therefore, at present, content ourself, by referring our readers to the first article, in the present number of the Journal, by the Syracuse editor, on the "privileges and duties of members," which are set forth in the writer's usual clear and forcible style. We hope this able and well written paper will be read by all. The truths which it embodies can hardly fail to be admitted by every one who will give it a deliberate perusal.—*Balt. Ed.*

*Misapprehension.*—Some persons, elected to membership in the American Society of Dental Surgeons, erroneously suppose, that by neglecting to pay their annual dues, they become honorary members. This is a mistake. None are so regarded by the constitution and by-laws, except those who are specially elected as such, and all who neglect to pay their dues for three years, by a regulation of the society, lose their membership. It would be well, therefore, for those who are in arrears to look to this matter, and, if they would retain their membership, to forward the amount of their dues to the treasurer, before the next meeting of the society, which is to take place the first Tuesday in August next.—*Balt. Ed.*

*Prepared American Oil Stone.*—In a former number of the Journal, allusion was made by the Washington city editor, to the great value of the above named stone to dentists and surgeons. It has, recently, been very greatly improved by the owner of the quarry, by a certain process through which he passes it, preparatory to use. The stone, as now prepared, cuts the instrument away much more rapidly, than when in the state from which it comes from the quarry, and puts an equally fine and delicate edge on it. Unlike other stones used for sharpening instruments, it is not liable to injury from sharpening pointed instruments, such as excavators, on it. It will retain a smooth, flat surface. Other stones are liable, when pointed instruments are sharpened on them, to become cut up in deep grooves or furrows.—*Balt. Ed.*

*Electro Magnetism.*—The employment of this influence is beginning to claim considerable attention as a remedial agent, especially in the treatment of neuralgic and rheumatic affections. We have known, in several instances, very happy effects to result from its use, and would recommend it to the profession as a valuable auxiliary in the treatment of those painful affections of the jaws, which cannot be traced to a diseased condition of the teeth, and for the relief of which, the dentist is oftentimes called upon to prescribe. It is becoming, we believe, very much in use by the Medical profession in the treatment of both acute and chronic diseases, and the reports of those who have employed it, speak strongly in its favor.

Our attention has recently been called to the subject by Dr. L. D. Fleming, of Newark, N. J., who has been employing it for some time with marked success. He has shown us some specimens of the instrument manufactured by S. B. Smith, of No. 115, Chambers-st., N. Y., which are got up in a very neat, cheap and portable form. Their power is perfectly at the control of the operator, and can be carried from an imperceptible impression to any that can be endured. The prices vary from fifteen to twenty dollars, according to the size and finish of the apparatus, and we believe they can be had as low as eleven or twelve dollars, but this size we should think a little too frail.

The instrument may be had of Dr. L. D. Fleming, 26 Walnut-st., Newark, N. J., or of J. M. Barstow, 3½ South 7th-st., Philadelphia.

[*Balt. Ed.*

*Quack Advertisements.*—It is an unpleasant, and generally a profitless task to either speak, write, or in any other manner lift the warning voice against that species of nuisance known as quack advertisements. We say, unpleasant is the duty of him who undertakes it, for if he discharges it properly, his conscience, and every principle of justice, honesty, and humanity, require that he should use the strongest, boldest and most uncompromising language of censure, and profitless, for the people in whose behalf he undertakes it, gives so little heed to the warning admonition.

But this forms no excusable ground for shrinking from the duty. Quack advertisements—to say nothing of the quacks themselves—we assert are the means of destroying daily an incalculable number of the lives, health and happiness of the people—that they have not a single element of good in them—but are only an evil, and that continually.

To guard therefore against their imposture, we will give, what we hold to be, the cardinal elements entering into their composition, and by which their true character may always be recognised and detected.

The *first most prominent element* is its CERTAIN INFALLIBILITY in every case, to the accomplishment of the object it proposes. Does it relate to the diseases of the human frame? The self-styled advertising physician asserts, without qualification, an infallible cure for the whole. Or if to a particular part of the body, as for example the teeth, an infallible, would-be-dentist gives most positive and unequivocal assurance that he can make, adjust, and replace teeth in a manner that can hardly be equalled by *nature herself*, but further, attempts even to put her to the blush.

*The second element* characterising quack advertisements, is, namely, their *unblushing impudence, falsehood and villainy*, for at a single sweep, they set aside and put at defiance all the accumulated knowledge of the learned, wise and good of the present and past ages, and falsely, unblushingly, and wickedly assert that the whole is nothing, and worse than nothing, in comparison with their own instinctive or improved, infallible and never-failing remedies.

*The third element* characterising quack advertisements is the universal ignorance or knavery of their authors.

Has any one been deceived? Let him inquire after the qualifications of his deceiver, and he will find him an illiterate, ignorant pretender, or otherwise an accomplished and designing knave.

These three elements constitute, we think, the great distinguishing marks of quack advertisements. We would therefore call upon the people as they value the beauty and soundness of their teeth—as they value their health, their happiness, and their lives, to seriously reflect upon the remarks we have just made, and we not only respectfully ask, but affectionately challenge, their honest reflection—and if they be true, then to shun all such advertisements as they would a viper—as they would their worst enemy. Quackery is always busy. It is never idle, neither by night nor by day; it is continually planning means by which to deceive the credulous and unsuspecting. Almost every newspaper in the country contains a glowing account of some wonderful and marvellous cure effected by some one of its thousand nostrums.

The foregoing remarks have been elicited by the frequency with which our attention has recently been called to the subject to which they refer.

We received a few weeks since a newspaper from Kentucky, containing one of the most fulsome and disgusting dental advertisements which we ever recollect to have seen. It covers about one-fifth of one of the pages of the paper, setting forth the pretensions of the author, *Dr. J. B. Stout,* and the marvellous virtues of his "Nervina Arcana, or *highly popular Nervines,*" in phraseology so strong, that it would seem presumptuous, almost in the highest degree, to doubt either the one or the other. Dr. S. is certainly a most wonderful man, at least in his own estimation, and if he cannot induce others to acknowledge his professional skill and profound abilities, he is determined that his own modesty shall not prevent him from proclaiming them. But the advertisement here alluded to, is not the only place in which he has endeavored to show himself off. We were shown, previously to our having seen this, a Philadelphia paper, containing an article under his signature, purporting to have been a lecture delivered before the American Society of Dental Surgeons, and in the heading of which, he is represented as a Vice President of that Association. The paper in question was never read before that body, nor has he ever been a member, much less, a Vice President of it.

But for the attempt at deception, last mentioned, we should not have felt ourselves called upon to notice Dr. Stout, but occupying the relationship to the American Society of Dental Surgeons we do, we feel called upon to make the above statement.

*Supernumerary Teeth.*—We are indebted to Dr. Townsend, of Philadelphia, for a plaster model of a most curious and remarkable example of supernumerary teeth. In the model, which was obtained from a wax impression of the superior dental arch, there are two molares,—the first and second, the dens sapientiæ not having yet appeared, three well developed bicuspides and two cuspidati on each side. There are also on one side of the medial line, two well formed lateral incisors and one central; on the other side, there is only the usual number—one central and one lateral.

[*Balt. Ed.*

*Porcelain Teeth.*—It was supposed, five years ago, that the *ne plus ultra* of perfection had been attained in the manufacture of porcelain teeth, but every year continues to add some improvement to this branch of the art. With regard to those manufactured by Mr. Stockton, they are too well known, and extensively used, to render it necessary to say any thing concerning them. The profession are, also, greatly indebted to Dr. Alcock of N. Y., for the very beautiful and excellent teeth with which he is furnishing them. But besides these two gentlemen, others are engaged in their manufacture. We have recently received some very fine specimens from Dr. Burr of Philadelphia, and Dr. Crane of Cincinnati.—*Balt. Ed.*

*New Postage Law.*—This goes into operation the first of July, and by its provisions, the postage on the Journal, hereafter, in any part of the United

States, will only be 8½ cents, instead of 25, as formerly charged. The reduction on letters is still greater, and in consequence of which, we hope to hear from our correspondents more frequently, and to have many new ones added to the list which we already have.—*Balt. Ed.*

*Professional Items.*—Dr. George W. Parmly, we are informed, proposes going to Europe in a few weeks, with a view, we believe, of making London the place of his future residence. Taking with him the high reputation he has acquired in this country, for skill as a practitioner of dental surgery, he could hardly fail of success any where. In him are united, in an eminent degree, both the high-minded gentleman, and scientific, skilful practitioner. . . . . . Mr. Addington, dentist of Norfolk, Va., sailed a few weeks since for Montevideo, South America, for the purpose of practising his profession in that place. . . . . . *Dr. E. Gidney.*—We are sorry to learn, that ill health has compelled Dr. Gidney to retire from the very lucrative practice which he has, for many years enjoyed, in Manchester, England. We are gratified, however, to be able to state, that he is about to return to the United States, and trust that he will here recover the health which he has lost abroad. We hope to have the pleasure of seeing him in August next, at the meeting of the American Society of Dental Surgeons, and of welcoming him back to his native land. Dr. G. has been one of the warmest friends of the Journal, having obtained for it a large circulation in Great Britain, and for which the Association, whose organ it is, owes him many thanks. . . . . . We announced some eighteen months or two years since the departure of Dr. J. R. Robinson, of Baltimore, for Valparaiso, South America. It now gives us pleasure to state, that we have subsequently heard of his safe arrival at that place, and that his success in practice there has equalled his most sanguine expectations. His brother, Dr. M. Robinson, sailed a few days since for the same place, with the view of joining him in practice. . . . . . Dr. C. O. Cone, late of Massachusetts, has recently moved to Georgetown, Ky. The eminent qualifications and skill in dental surgery possessed by Dr. C., will, we feel assured, secure for him the confidence and patronage of the citizens of that place. We know him to be both a good theorist and good practitioner. . . . . . Dr. J. B. Savier, known to the profession as the translator of Gariot on the Diseases of the Mouth, and Maury's Dental Surgery, has moved to Montgomery, Alabama. We wish him success in this new field which he has selected for his professional labors. . . . . . We are gratified to learn that Dr. N. A. Fisher, of Providence, R. I., who went to the south last winter, for the benefit of his health, has returned, with it greatly improved.—*Balt. Ed.*

